# InspirE5: a participatory, internationally informed framework for health humanities curricula in health professions education

**DOI:** 10.1186/s12909-022-03551-z

**Published:** 2022-06-24

**Authors:** Sandra E. Carr, Anna Harris, Karen Scott, Mary Ani-Amponsah, Claire Hooker, Brid Phillips, Farah Noya, Nahal Mavaddat, Daniel M. Vuillermin, Steve Reid, Pamela Brett-MacLean

**Affiliations:** 1 Health Professions Education, University of Western Australia, Perth, Australia; 2 Faculty of Arts and Social Sciences, Maastricht University, Maastricht, Netherlands; 3 Medical School, University of Sydney, Sydney, Australia; 4 College of Health Sciences, University of Ghana, Accra, Ghana; 5 Medical School, University of Pattimura Indonesia, Nusaniwe, Indonesia; 6 Medical School, University of Western Australia, Perth, Australia; 7 Institute for Medical Humanities, Peking University, Beijing, China; 8 University of Cape Town, Cape Town, South Africa; 9 Faculty of Medicine & Dentistry, University of Alberta, Edmonton, Alberta Canada

**Keywords:** Health humanities, Curriculum, Evaluation, Health professions education, Medical humanities

## Abstract

**Background:**

Reporting on the effect of health humanities teaching in health professions education courses to facilitate sharing and mutual exchange internationally, and the generation of a more interconnected body of evidence surrounding health humanities curricula is needed. This study asked, what could an internationally informed curriculum and evaluation framework for the implementation of health humanities for health professions education look like?

**Methods:**

The participatory action research approach applied was based on three iterative phases 1. Perspective sharing and collaboration building. 2. Evidence gathering 3. Development of an internationally relevant curriculum and evaluation framework for health humanities. Over 2 years, a series of online meetings, virtual workshops and follow up communications resulted in the production of the curriculum framework.

**Results:**

Following the perspective sharing and evidence gathering, the *InspirE5* model of curriculum design and evaluation framework for health humanities in health professions education was developed. Five principal foci shaped the design of the framework. *Environment*: Learning and political environment surrounding the program. *Expectations:* Graduate capabilities that are clearly articulated for all, integrated into core curricula and relevant to graduate destinations and associated professional standards. *Experience:* Learning and teaching experience that supports learners’ achievement of the stated graduate capabilities. *Evidence:* Assessment of learning (formative and/or summative) with feedback for learners around the development of capabilities. *Enhancement:* Program evaluation of the students and teachers learning experiences and achievement. In all, 11 Graduate Capabilities for Health Humanities were suggested along with a summary of common core content and guiding principles for assessment of health humanities learning.

**Discussion:**

Concern about objectifying, reductive biomedical approaches to health professions education has led to a growing expansion of health humanities teaching and learning around the world. The *InspirE5* curriculum and evaluation framework provides a foundation for a standardised approach to describe or compare health humanities education in different contexts and across a range of health professions courses and may be adapted around the world to progress health humanities education.

## Background

Recent trends have revealed an increasing presence of the humanities in the education of students from the health professions prior to their registration to practice. A broad interdisciplinary field, ‘health humanities’, encompasses perspectives, insights and approaches from diverse arts (e.g., visual arts, performing arts, music), humanities (history,literature/narrative, ethics and philosophy) disciplines and the social sciences (sociology, anthropology) [1–3]. As stated by Shapiro ( [4], p.192], the educational aim is to help health professions students “better understand and critically reflect on their professions with the intention of becoming more self-aware and humane practitioners” [4].

Teaching programs in the health humanities, mostly located in medical and nursing schools, have been established all around the world since the late 1990s, with growing mainstream acceptance of their value over the past decade [5, 6].

And yet even as the popularity of these programs is growing, they remain structurally marginal and held back from their potential for transformative impacts. Much of the development in research about health humanities for education appears to rely on committed individuals and teaching of supplementary subjects. They are very rarely, if ever, integrated and part of core curricula [7]. This is reflected in the developing evidence base for the health humanities in health education. This is mostly composed of small, heterogenous and variegated case studies. Measuring and quantifying the impact of health humanities education has proven challenging because courses are diverse in their content, goals, methods and assessments. A recent review by Moniz in 2021 concluded that the literature surrounding health humanities in health professions education is characterized by descriptions of brief, episodic instalments [8].

Perhaps most importantly, there is a continued absence of a bigger vision for the health humanities, one that can provide a paradigm for research, development and assessment adequate to the challenges facing twenty-first century healthcare professions education. Moniz’ review reflected this: most studies favoured a biomedical orientation, and were largely lacking the application of theoretical frameworks that may support accumulation of evidence into a bigger picture view of health humanities education [8]. Some scholars have provided some ideas for how the field might develop. Alan Bleakley, for example, has discussed how, if the health humanities were truly integral in health professions education as core curricula, they could re-integrate with the inevitably reductive forms of biomedicine and produce an education that is authentically centred on caring for the ‘person in context’. [7] Health Humanities for education was envisaged by Dennhardt in a systematic review, as aiming to enable students to explore a ‘values-oriented’ education as the foundation of becoming a health professional [9]. Others have argued that health humanities education engages students in critical thinking, allowing them to grasp the complexity of human contexts of health and healthcare, promoting the value of diverse perspectives in understanding the human consequences of health and illness [4, 6].

Also missing are processes for developing a wider international perspective for health humanities. There is a pressing need to explore health professions and health humanities educational landscapes with the purpose of appreciating the range of intersectional contextual lenses (historical, cultural, geographical, political, structural, etc.) needed to ensure an internationalist perspective that can progress toward social justice, digital health and increasingly, our planetary health. There are some published manuscripts articulating differences in approaches taken, for example comparing China and western countries [10] and a growing number of networks to facilitate discussion across continents such as Africa and more globally [11, 12]. However, a review of the health humanities educational landscape with the purpose of informing a more internationally relevant curriculum is becoming a priority.

Against this backdrop, a team of health humanities educators, scholars and practitioners from culturally diverse settings came together in 2019 through the Worldwide Universities Network (WUN). The intention was to facilitate reporting on the effect of health humanities teaching in undergraduate health professions education courses to facilitate sharing and mutual exchange internationally, and the generation of a more interconnected body of evidence surrounding health humanities curricula. The intended purpose of the work included offering course-accrediting bodies an approach to standardise the description of health humanities, through a collective vision statement and inclusive of suggested learning outcomes and curriculum content, teaching strategies and assessment of learning. The countries represented in this WUN Health Humanities initiative are Australia, Canada, China, Ghana, Indonesia, South Africa and The Netherlands.

In this paper we detail the participatory action research (PAR) approach we followed which included iterative cycles of reflection, data collection, and action [13, 14]. This was well-suited to our needs as we sought to create an opportunity for open dialogue and meaningful discussion and develop a rationale for inclusion of health humanities as core curricula in health professions education. Our approach was based on the assumption that educators, scholars, and healthcare professionals who work within, or in support of, health humanities for health professions education were well-positioned to develop a guiding curriculum framework in a collaborative and participatory manner [15]. Our overarching aim was to facilitate a range of ideas and contributions regarding what an internationally informed curriculum and evaluation framework for health humanities in health professions education might look like [16, 17].

## Methods

This project was undertaken over a period of 2 years, (July 2019 to August 2021) by a research team of 11 that included one health professions educator with a clinical background as a midwife,(SC) two family physician academics (SR, NM), two medical educators (FN, KS), one nursing academic (MAA), an arts-based health humanities scholar (CH), a medical /health humanities educator and scholar(interdisciplinary studies, arts and health) (PBM), one doctor turned anthropologist of medicine/medical education (AH), and two lecturers in health humanities, one with a background in nursing and English and cultural studies (BP) and the other a background in history (DV). All have engaged with health humanities in the education of health professionals over a number of years. The team deliberately included senior academics and early career academics with two PhD students and purposefully formed a collaboration of stakeholders with a more diverse international representation than is represented by the literature on medical and health humanities. We adopted an approach that emphasises collective inquiry grounded in the experience of the participants [15, 18]. As ‘insider researchers’ we were aware of the need to be reflexive and cognisant of how one’s assumptions might differ from those of others who are not included in the work [19]. The diversity of the group’s background and experience meant we often viewed information from a range of perspectives which promoted participation, active discussion and collaboration before reaching any agreement.

Once the initial collaboration was formed in mid-2019 (SC, AH, CH, SR, PB-M), funding was sought and obtained in December 2019 from the Worldwide Universities Network (WUN) as part of the Global Higher Education and Research challenge. The original plan was for the group to meet in person on two occasions over the 2 year period with online meetings during intervening periods. However travel was not possible with COVID-19 in 2020 and 2021 so the project was conducted via virtual meetings. In some ways, this meant the group was able to meet more frequently with shorter periods between each meeting which allowed for an ongoing iterative cycle of reflection. Ethical approval for the project was not required.

There were three phases to the project that was primarily completed through a series of seven online meetings lasting 60 to 90 minutes, two virtual workshops and 3 months of follow up email communications with comments on the versions of output reports and documentation. Each meeting and workshop included sending the project team notes of the meetings with reflective prompts and actions during the intervening period. The activities of project team through the three phases are outlined below. The purpose and outcomes of the meetings and workshops are summarised in Table 1.

1. *Perspective sharing and collaboration building*
  As the project team did not previously know each other well, the purpose of this phase was to form the group so as to enable the achievement of productive and collaborative work. The outcomes of this phase were a protocol for a scoping literature review on the Health Humanities and a structured written reflective activity on the project team’s international perspectives. The written reflections were framed around two central questions: ‘*What learnings are there from the differences and juxtaposition of international perspectives?’* and ‘*What does it mean to think about health humanities from a more global perspective’?* The written reflections were combined and reviewed to provide a shared understanding of health humanities for the project team [20].
2. *Evidence gathering*
  The purpose of this second phase was to gather evidence so as to identify the foci of health humanities teaching and inform the development of the curriculum framework. Given previous systematic reviews of the literature had focussed on quantitative research [8, 9, 21, 22], we conducted a focused scoping review of qualitative and mixed-methods studies that included the influence of integrated health humanities curricula in pre-registration health professions education with program evaluation of outcomes. The scoping review was undertaken over 8 months and the findings were integrated with those previously identified through quantitative research [2].
3. *Development of an internationally informed curriculum and evaluation framework for health humanities*
  Two, 3-h virtual workshops run 3 days apart (see Table 1), along with three subsequent virtual meetings focused the development and guiding structure for the framework and the identification of the suggested graduate capabilities for health humanities education in the health professions. The final drafts of the curriculum and evaluation framework were completed once the draft framework was presented at the Association for Medical Education in August 2021 [23] and the scoping literature review was published [2].

**Table 1:** Project team activities, by phase - all online/virtual (2020-21)

Phase^**a**^	Date(s)	Type	Aim/Activities	Outcomes
1, 2	Feb 26, 2020	Project MTG 1 (1.5 hrs)	• Introductions, review project plan (timeline, team member contributions) • Discuss draft protocol for scoping review	• Confirmed interest of invited team members; recognized need to increase international representativeness of the team (beyond Australia, Canada, South Africa, and the Netherlands) - additional collaborators invited to participate via the World University Network (WUN) • Decision to include research across ALL pre-registration health professions students (medicine, dentistry, nursing, allied health); refocused review towards how and why Health Humanities (HH) are used in health professions education (focus of teaching and learning; domains and levels of learning), and how HH curriculum is evaluated.
1, 2	Apr 7, 2020	Project MTG 2 (1.5 hrs)	• Introduce new team members from China, Ghana, & Indonesia • Review revised scoping review protocol	• Expanded international scope of project team • Scoping review protocol finalized
1, 2, 3	May 21, 2020	Project MTG 3 (1 hr)	• Discuss approach for sharing diverse perspectives on HH • Report on progress of scoping review • Discuss approach for developing a draft curriculum/evaluation framework	• Two 2-hour workshops scheduled (Jul 21 & 24, 2020); team members to submit written reflections on “*What Health Humanities means to me”* (500-700 words) • Initial search netted 8594 article records (an additional 27 articles were also later identified)
1, 2	Jul 7, 2020	Project MTG 4 (1 hr)	• Confirm workshop for sharing diverse perspectives on HH • Report on progress of scoping review	• Team members to submit written reflections on ‘*What learnings are there from the differences and juxtaposition of international perspectives?’,* and ‘*What does it mean to think about Health Humanities from a more global perspective?’* • Screening of 8606 article titles completed with 410 articles identified for abstract screening; data extraction approach confirmed
1, 3	Jul 21, 2020	Project Workshop (1/2) (3 hrs)	• Discuss perspectives on HH • Further discussion re: approach to curriculum/evaluation framework	• Common concepts associated with HH identified and shared across the project team • Stake’s (2003) responsive evaluation model to guide development of a framework
3	Jul 24, 2020	Project Workshop (2/2) (3 hrs)	• Further discussion re: curriculum/evaluation framework	• Social constructivism identified as the dominant education theory, along with reflective learning theory; confirmed focus on *capabilities* in place of competency-based learning outcomes
1, 2, 3	Aug 26, 2020	Project MTG 5 (1.5 hrs)	• Discuss preliminary draft of a shared global vision for HH • Report on progress of scoping review • Further discussion re: approach to curriculum/evaluation framework	• Shared global vision statement drafted • Screening of 410 article abstracts completed; 71 articles identified for full manuscript review • Agreement on 11 graduate capabilities; range of assessment strategies identified; preliminary draft of curriculum and evaluation framework to be circulated for feedback
2, 3	Dec 8, 2020	Project MTG 6 (1.5 hrs)	• Report on progress of scoping review • Discuss draft curriculum and evaluation framework • Plan for sharing findings (beyond funding period)	• Data extracted from final set of 24 scoping review articles; first draft of results written • Further development, revision of curriculum and evaluation framework • Conference abstracts submitted; extension of funding from WUN requested
1, 2, 3	Feb 11, 2021	Project MTG 7 (1.5 hrs)	• Discuss ideas for article on HH for an interconnected world • Discuss preliminary draft of scoping review paper • Discuss plans for paper on the proposed HH curriculum and evaluation framework	• Support for drafting a “perspective” article on HH considered within a global context • Feedback on first draft of scoping review manuscript • Proposed draft HH curriculum and evaluation framework finalized
1, 2, 3	Mar-Aug, 2021	Regular Email Communication	• Feedback on presentations planned for ANZAHPE and AMEE • Manuscript preparation (contributions, consultation, feedback) - perspective article, scoping review, framework paper	• Conference presentations completed; proposed HH curriculum and evaluation framework finalized • All first drafts of manuscripts completed

^a^Phase: 1 - Perspective sharing & collaboration building; 2 - Evidence gathering via scoping review; 3 - Development of an internationally informed curriculum and evaluation framework for Health Humanities

## Results

### Phase 1: perspective sharing and collaboration building

#### Opening to a juxtaposition of perspectives: possibilities for an international perspective of health humanities

Through the discussions and written reflection we agreed that the Health Humanities ‘holds space’ for dialogue between health and biomedical research and practice on the one hand, and the humanities, arts and social sciences on the other. Since these respective ways of thinking have developed from quite different paradigms, languages, traditions and norms, a core activity in health humanities education is the constant work of ‘translation’. This work is of course necessary for all involved in bridging the disciplinary divides. Alternatively, we recognised that it is easy to get into a narrow mindset when only engaging with local partners in designing health humanities education, because potentially challenging issues such as assessment and interdisciplinary practice need a broad perspective. Important elements of the international perspectives included open meaningful dialogue reflecting on different perspectives, which we found offered more diversity in social, cultural, geopolitical, and humanities contexts for our study [20].

#### Health humanities from a more global perspective

A common thread signposted was that holism or having a holistic outlook is relevant to many perspectives around health humanities education. Our collaboration also led us to formulate pathways to navigate the tension between localisation and the dominant modes of health humanities dialogue. We suggest that international collaborations and networks may be the way forward for others in the field too [20].

There was also an impetus within the group, particularly from the perspective of low and middle income countries, to consider how health humanities can be utilised to bring advocacy into health professions education [24]. An activist approach is needed to confront global health issues such as poverty, climate change, educational and health inequalities and ethical issues such as the rise of technology in health care [20]. An international outlook on the Health Humanities can unpack approaches to these problems which are both complex and interconnected, and which benefit from international comparison and sharing (see Table 2).

**Table 2:** International view point for health humanities education

*Internationally applicable approaches to health humanities curricula include learning that supports reflection, critique, and consideration of personal values and beliefs. This in turn enhances an orientation to, and understanding of others (perspective taking, advocacy, empathy and other relational capabilities) and supports responsiveness and commitment to collaborative action directed at humane healthcare of significant local, international and global health issues*	

### Phase 2: evidence gathering

The literature search, which covered a 5 year period (2015-2020), identified 8621 publications with 24 articles meeting the criteria for inclusion. Over half of these were published in North America (*n* = 13); the remaining were based in England, Ireland, Australia, India, New Zealand, Spain and Sweden. Only the one from India was not from a high income country [25]. Many reported health humanities curricula focused on developing students’ capacity for perspective, reflexivity, self-reflection and person-centred approaches to communication however learning outcomes were not consistently described. The primary finding of the review was that at present, there is an absence of a consistent framework for health humanities learning, teaching and assessment, and hence, little capacity for systematic evaluation within or across curricula. The findings recommended the need to articulate a more systematically realised and empirically informed set of core capabilities for health humanities that can be adapted for local educational contexts. The value of core capabilities for developing health humanities curriculum within a programme was reported as being able to more systematically develop integrated learning activities that can achieve some of the higher-order educational outcomes and more accurately and systematically evaluate whether these core capabilities are being achieved and thereby inform the development of a curriculum framework [2].

### Phase 3: curriculum and evaluation framework for the health humanities

For health humanities curricula to become integrated as core, some key antecedents and facilitators for success require consideration [26]. These include the external influences that may be outside of course coordinators’ control but can impact successful curriculum implementation. For example, the AAMC states that it strongly endorses all medical schools to offer some medical humanities-focused learning [27]. Similarly, the Health Professions Council of South Africa stipulates a key competency as being a patient/client-centred approach [28]. Likewise, academics in programs of healthcare management are calling for a dialogue surrounding the utility of humanities to broaden the scope of required competencies of their accrediting bodies [25]. This recognition and validation by an external or accrediting body acts as evidence in supporting the validity of health humanities education and is useful when looking to obtain support from local leadership and inspire engagement from within an institution. The development of the curriculum and evaluation framework recognised this need for some impetus or inspiration when proposing the introduction of a Health Humanities curriculum. The design of the framework focused on some key aspects of curriculum design as summarised in Table 3 [29], to develop the ***InspirE5*** model of curriculum design for Health Humanities.

**Table 3:** *InspirE5* model of curriculum design for Health Humanities

**Inspire:** identify internal and external influencing factors that can inspire the development, implementation of and commitment to Health Humanities curriculum in health professions education	
A. Environment: Learning and political environment surrounding the program including antecedents, facilitators and barriers.	
B. Expectations: Graduate capabilities that are clearly articulated for all, integrated into core curricula and relevant to graduate destinations and associated professional standards.	
C. Experience: Learning and teaching experience that supports learners’ achievement of the stated graduate capabilities.	
D. Evidence: Assessment of learning (formative and/or summative) with feedback for learners around the development of capabilities.	
E. Enhancement: Program evaluation of the students and teachers learning experiences and achievement.	

#### Environment: learning and political facilitators and barriers to be aware of

When introducing health humanities teaching, it is helpful to be aware of the curriculum model or models informing the overall educational program, and array of instructional methods used (problem-based learning, interprofessional, integrated community-based learning, etc.), as well as recognized gaps, to identify opportunities for interweaving humanities offerings and helping learners appreciate connections with other aspects of the curriculum [30, 31]. Others have also emphasised the need for this unifying perspective in curriculum design that focuses upon construction and maintenance of a particular learning climate [7, 31]. Imperative for success is having academic staff who are confident to apply innovative teaching strategies or inspire others and make the most of opportunistic curriculum innovation and can be the pioneers or the champions. We recognised that bringing health humanities teachers and researchers together in a collaborative way such as at health professions education conferences and through networks may support faculty and curriculum development. It is also important to recognise that health humanities can be perceived as competing with other disciplines in what is typically a time and resource poor environment. Finding ways to work within an existing curriculum can make it easier for the learning experience to be integrated rather than stand alone and makes it easier to prevent competition or obstruction but does require appropriate faculty development. Our collective experience and findings of the scoping review [2] suggests that persistence of supporting people who are strengths-based with a collaborative orientation, can successfully combat individualistic academic norms. Tapping into areas of possible student dissatisfaction with the biomedical model of education or the learning environment can act as the impetus or inspiration for curriculum renewal and inclusion of health humanities.

#### Expectations: graduate capabilities that are clearly articulated for all

The research group determined to focus on graduate capability, rather than competence as better preparing health professionals to respond to the challenges of working in the contemporary international health sector [32]. Capability is the ability to adapt to change, generate new knowledge, and continuously improve performance [28], whereas competence-based approaches are less dynamic, focus on the current state, having the knowledge and skills necessary to perform a job [32, 33]. Capability embeds the integration and adaptation of knowledge, skills and personal qualities through scaffolded learning that enables generation of new knowledge and adaptation to change and uncertainty [33, 34]. We propose graduate capabilities that align with learning and teaching strategies and topics commonly reported in health humanities literature, as outlined in Table 4.

**Table 4:** Suggested Graduate Capabilities of Health Humanities Curriculum for Health Professions Education

By engaging in Health Humanities education, students will develop capability to:	
**Observe astutely** - *have or showing an ability to notice and understand things clearly*	
**Self- Reflect** – *capacity to exercise introspection*.	
**Appreciate ambiguity** – *able to deal with increasing uncertainty*	
**Collaborative Critic** – *use a community approach to examine and potentially produce better understanding of a problem or situation*	
**Practise Evidence Synthesis** – *bring together all relevant information on a research question to identify gaps and establish an evidence base for best-practice guidance*	
**Engage in Dialogue** – demonstrate *capacity for an exchange of ideas* via *communication with others*	
**Interpret Perspectives** – *to look beyond one’s own point of view, to consider others’ thoughts, opinions and feelings about something*	
**Value the Narrative** - *to be grounded in the reality of the present and illuminate the reality of the past.*	
**Value Person-centredness** – *put the interests of the individual receiving care or support at the centre of thoughts and action*	
**Appreciate innovation** – *an enduring capacity to change and improve*	
**Relational Responsiveness-***able to recognize interconnectedness with others, and respond in relation to positive possibilities for going forward*	

#### Experience: learning and teaching experiences

The interdisciplinary nature of health humanities sees learning occurring at multiple intersections between a range of humanities disciplines and the health and medical sciences. The humanities disciplines frequently referred to in published studies include philosophy, sociology, literature, visual arts, music, narrative and performing arts and less frequently but equally valuably, history (in context and place, for example China, Africa, Australia) and anthropology [2]. This current work identified how health humanities teaching strategies are being applied to emergent issues for health professions education such as management of high stress work environments and burnout for health professionals, responses to the COVID-19 pandemic and the utility of technology for healthcare. From our collective experience and review of the literature the common content topics being taught as part of health humanities curricula can be summarised as depicted in Table 5.

**Table 5:** Common content covered in health humanities education

Origins of Values & Beliefs	Ethical Reasoning	Legal Principles	Empathic Communication	Compassion
Advocacy	Systems- Complexity	Tolerance of Ambiguity	Appreciation of Diversity (Gender, culture, spirituality)	Exploring experiences of Health
Exploring Health Care Systems	Person Centeredness	Evidence Synthesis	Research Paradigms	Research Skills (Qualitative)
Arts in Health	Reflective Practice	Collaborative Practice	Self-Care as a Health Professional	Professional Behaviour
Climate Change response	Sustainability of Health Care	Health Literacy	Technology – digital humanities	Social Justice

Health humanities focused education is reported to enable students, through engaging learning and assessment experiences that are predominantly in face to face settings, to create a learning environment that encourages students to reflect, critique and consider their personal values and beliefs. The teaching methods encountered are most often small group in nature, whether delivered in online or face-to-face modes, and involve sharing of thought and reflections in dialogue with peers and mentors. This learning commonly aims to enhance the future health professionals’ capability for self-reflection, advocacy, empathy, leadership, followership, scholarship and person centred communication [2, 8, 20, 21].

#### Evidence: guiding principles for assessment of health humanities and assessment strategies

Through discussion and review of the literature, the project team identified three apparent guiding principles surrounding the assessment of student learning in health humanities. Firstly, the approaches to assessment often expected the students to *engage in the act of creation* to demonstrate achievement of a health humanities capability. This creation was often a written piece (essay, narrative, story, and reflection) or an object (concept plan, drawing, picture, sculpture, painting) or a performance (art, music, theatre) and sometimes included the application of technology (blog, podcast, video). Secondly there was always an engagement with the object created through reflection and the *articulation of reflective thought.* Finally, the assessment commonly *explored values and beliefs* of the students. This was sometimes to identify values or to understand the presented values and beliefs and thereby enhance capacity for divergent perspectives. On other occasions the assessment focused on shifts in values towards professionally accepted standards. The assessment of achievement of the graduate capability utilised critical evidence synthesis, self- assessments, peer assessment, direct observation of performance and work integrated assessment of professional practice rather than objective, measurement based assessments. Assignments were more frequently used than examinations.

#### Enhancement: program evaluation of student and teachers learning experiences

Responsive evaluation is an approach that places importance on quality improvement and the representation of quality in a program rather than just the educational outcomes achieved [26]. When compared to other evaluation approaches, it draws attention to program activity, program uniqueness, and the social plurality of the people running a program [35]. This responsive approach is applicable to summative and formative evaluations and allows for flexibility and ambiguity. Formative evaluation is useful when staff need help monitoring a program and when problems are difficult to identify and articulate. Summative evaluation is useful when audiences want to understand the activities, strengths and shortcoming of a program [35]. In responsive evaluation, data is gathered around the processes and activity of a program using quantitative and qualitative methods. We chose a responsive model to curriculum evaluation of health humanities programs because of this recognised capacity for tolerance of ambiguity. That is, we recognised that internationally, health humanities education programs in the health professions are often not mainstream programs, there can be a lack of clarity about success indicators, they are usually collaborative and multidisciplinary, making their implementation complex and sometimes ad-hoc in nature, resulting in an absence of consensus of purpose and method amongst stakeholders [36]. Based on Kirkpatrick’s levels of evaluation [37], the scoping review of health humanities and other systematic reviews, and informed by other published evaluation matrices, we designed the evaluation matrix depicted in Table 6 for use by educators of health humanities in health professions education [38].

**Table 6:** Enhancement: evaluation matrix for health humanities curricula in health professions education (Adapted from Gibson et al., 2008) [38]

*Health Humanities Program Quality Aspects*	*Component*	*Key Quality Indicators*
Student Wellbeing	SW1. Engaged and supported	1.Student engagement with learning 2.Student access of support and wellbeing services 3.Student perception of support services provided by institution 4.Student academic progression
Learning Environment	LE 1.Curriculum	1.Staff perception of facilitators and barriers for change 2.Past program evaluation and accreditation reports of learning environment 3.Political environment supporting health humanities curricula
Student Experience (SE)	SE1. Learning & Teaching	1.Student Satisfaction with learning and teaching (includes activities) 2. Student perception of quality of learning and teaching materials 3. Student perception of quality of physical environment 4. Student perception of quality of learning culture
	SE2. Administration & Support	5. Satisfaction with administration and support
	SE3. Sense of Community	6. Quality of interactions and support
Student and Graduate Capabilities (SG)	SG1 Student Capabilities	1. Assessment results profile 2. Progression of students
	SG2. Graduate Capabilities	3. External capability assessments 4. Perceived self-efficacy
	SG3. Application of capabilities to clinical practice	5. Paths, diversity, achievement of graduates
Staff & Teaching (ST)	ST1 Merit and capabilities	1. Qualifications and experience 2. Scholarship of teaching
	ST2. Support, development & recognition	3. Career development, support and workload management
	ST3.Quality of Teaching	4. Student satisfaction with learning and teaching 5. Alignment of teaching, learning and assessment- document review 6. External assessment of quality of teaching
Curriculum & Resources (CR)	CR1 Quality of curriculum design	1. Stakeholder judgements of quality of curriculum design 2. Evaluation and improvement processes informing change
	CR2. Curriculum ownership and sustainability	3. Program engagement and commitment
	CR3. Suitability of resources	4. Suitability of resources (physical, ICT, materials)

## Discussion

This project offers some insight from an international perspective, into what a curriculum and evaluation framework for the implementation of health humanities for health professions education could look like. This paper also provides an illustration of how participatory action research can be applied in the area of curriculum and evaluation development. Our framework ***Inspir*E5,** provides a means to respond to the visions for how the health humanities could be integrated in core curricula such that values-based professionalism could be cultivated [18] critical thinking and complexity perspectives honed [39] and care and sensibility re-centred in moments and practices that either resist or re-connect, biomedical reductivism or healthcare hierarchies [7, 9].

Most importantly, ***Inspir*E5** offers a way of considering the graduate capabilities of health humanities, the common health humanities content and some recurring guiding principles of assessment of learning in ways that are adaptable to local contexts. It is our hope that this framework offers a point of reference when designing learning and teaching activities for health humanities. While it is recommended that the graduate capabilities are selected based on the priorities of a particular context, it is not expected that all of the capabilities will be addressed or assessed.

The accompanying enhancement evaluation matrix (Table 6) offers a way of considering the process and outcomes of health humanities educational interventions in a way that facilitates a responsive approach to iterative curriculum enhancement. This curriculum and evaluation framework may also offer education researchers some common ways to report and compare health humanities education across programs and between institutions, even while acknowledging the specificities of their own cultural contexts. The next steps from this work, includes exploring opportunities for the WUN Health Humanities Initiative to use the ***Inspir*E5** framework to describe and audit the health professions education courses offered at the partner institutions and indeed other WUN partner universities. This would enable opportunities to document the relevance of the curriculum framework with other health professions educators in different contexts and across a range of health professions courses. Other outcomes of having this ***Inspir*E5** framework for health humanities curriculum includes the opportunity to conduct research exploring the achievement of the graduate capabilities or changes in learners achievement of the graduate capabilities in health humanities. Alongside curriculum development, the ***Inspir*E5** framework could also be used to structure faculty development in health humanities education that would support the integration of health humanities within ongoing learning environments.

Throughout this PAR we all knew in advance, but discovered more about and better recognised the paradox of trying to design and implement an international framework, or a global core curriculum in the health humanities, while centering the diversity of contexts of health humanities, teaching and practice. But working with these productive tensions is both creative and exciting: we suggest that educators and scholars around the world, will find this account of our experience and recommendations for Health Humanities curricula and evaluation a catalyst or the inspiration for new design, research and teaching. We offer our view that systematic approaches to assessment will support our aims for integrated health humanities teaching in health professions education, while remaining sensitive to and adapted for local contexts.

## Limitations

While we sought to develop a curriculum and evaluation framework with international relevance, the project team can only represent health humanities as they have experienced it. Therefore the main limitation of the study is that there may be aspects of the framework that are not useful for some or generalizable.

## Conclusion

This work illustrates how it was possible, even during a pandemic, to bring together a collaboration of academics who did not previously know each other, from a variety of backgrounds and universities to develop a health humanities curriculum and evaluation matrix that may be adapted around the world to progress health humanities education. This work has provided a foundation for our shared vision of healthcare that is always reflexively centred on social justice and person-centred care through the challenges of the twenty-first century.

## Data Availability

All data supporting the findings are represented in the paper in summary form. Further access to that data are however available from the authors upon reasonable request.
